# Randomized controlled trial of standardized *Bacopa monniera* extract in age-associated memory impairment

**DOI:** 10.4103/0019-5545.31555

**Published:** 2006

**Authors:** Sangeeta Raghav, Harjeet Singh, P.K. Dalal, J.S. Srivastava, O.P. Asthana

**Affiliations:** * Senior Medical Officer, Peninsula Community Mental Health Service, Frankston Hospital, Frankston, Victoria, Australia; ** Professor, Department of Psychiatry, KGMU Lucknow, Uttar Pradesh; *** Professor, Department of Psychiatry, KGMU Lucknow, Uttar Pradesh; **** Scientist F, Division of Clinical and Experimental Medicine, Central Drug Research Insitute, Lucknow; ***** Head, Division of Clinical and Experimental Medicine, Central Drug Research Insitute, Lucknow

**Keywords:** Brahmi, *Bacopa monniera*, cognition, memory, AAMI

## Abstract

**Background:**

Brahmi (*Bacopa monniera*) is a traditional Indian medicinal plant which causes multiple effects on the central nervous system. The standardized extract of this plant has shown enhanced behavioural learning in preclinical studies and enhanced information processing in healthy volunteers.

**Aim:**

To study the efficacy of standardized *Bacopa monniera* extract (SBME) in subjects with age-associated memory impairment (AAMI) without any evidence of dementia or psychiatric disorder.

**Methods:**

A double-blind, placebo-controlled randomized study design was employed. The subjects received either 125 mg of SBME or placebo twice a day for a period of 12 weeks followed by a placebo period of another 4 weeks (total duration of the trial 16 weeks). Each subject was evaluated for cognition on a battery of tests comprising mental control, logical memory, digit forward, digit backward, visual reproduction and paired associate learning.

**Results:**

SBME produced significant improvement on mental control, logical memory and paired associated learning during the 12-week drug therapy.

**Conclusion:**

SBME is efficacious in subjects with age-associated memory impairment.

## INTRODUCTION

*Bacopa monniera* (Brahmi) is an Ayurvedic medicinal plant, traditionally described with memory-enhancing, analgesic, sedative, anxiolytic and antiepileptic properties. The standardized *Bacopa monniera* extract (SBME), developed in the Central Drug Research Institute, Lucknow, has been documented to facilitate acquisition, consolidation and retention of newly acquired behavioural responses in animal models using three learning schedules.^1^ The amnestic effects of scopolamine, convulsive shock and behavioural stress were also reversed with *B. monniera* extract.^2^ The active constituents were identified as bacosides A and B.

The preclinical studies supported the two open clinical studies reporting enhanced memory and learning effects with *B. monniera* in children^3^ and patients with anxiety state.^4^ These studies have inherent methodological weaknesses as they lack a double-blind design, placebo control, randomization and evaluation on a valid neuropsychological battery of tests. In a recent double-blind placebo-controlled trial, the extract was shown to enhance information processing in healthy volunteers on long-term administration.^5^ The study used a double-blind, placebo-controlled design. Forty-six healthy volunteers received the extract for 12 weeks and the results showed improved cognitive processing including learning rate and memory consolidation measured by auditory verbal learning test. Though the exact mechanism of action of *B. monniera* is not known, there is evidence of its action on the cholinergic system. The effect of *B. monniera* includes modulation of acetylcholine release,^6^ choline acetylase activity and muscarinic receptor binding.^2^ Further, *B. monniera* has also been shown to have potent antioxidant properties,^7^–^8^ which reduce the oxidative stress in the ageing brain and may help in reducing amnestic effects.^9^ These preclinical evaluations were followed by toxicity studies including subacute, chronic, mutagenicity and teratogenicity which showed it to be safe in employed dose ranges.

In view of the above preclinical evidence of efficacy along with improved higher cognitive processes following information input from the environment, leading to enhanced learning/memory in clinical situations, the present study was carried out to evaluate the effect of SBME in subjects with age-associated memory impairment (AAMI). Memory is a complex function and ageing causes deterioration of various aspects of memory performance in normal adults.^10^ The older healthy subjects who are not demented but complain of memory problems and exhibit symptoms of memory loss in daily life, are familiar to clinicians all over the world. Kral introduced the term ‘benign senescent forgetfulness’ for this condition.^11^,^12^ However, Kral did not operationalize this concept. To characterize this phenomenon more precisely, a National Institute of Mental Health work group was formed that proposed research criteria for AAMI.^13^ This study reports effects of SBME in subjects with AAMI.

## METHODS

Subjects above 55 years of age with complaints of memory impairment were recruited by announcement through radio programmes, advertisements in local newspapers and by distributing handbills. These subjects had memory loss in everyday activities—difficulty in remembering names of individuals following introduction, misplacing objects and difficulty in remembering telephone numbers. Subjects with logical subset score <6 (Wechsler Memory Scale)^14^ were included but those scoring >24 on Mini Mental State Examination^15^ were excluded from the study. Each subject was required to execute written informed consent to participate in the trial, which was approved by the Institutional Ethics Committee of King George Medical College (KGMC), Lucknow, Uttar Pradesh.

A double-blind, placebo-controlled, parallel randomized design was used. Subjects received either standardized *B. monniera* extract (125 mg) or a placebo in twice a day schedule for 12 weeks, followed by the placebo for another 4 weeks to both the groups, making 16 weeks as the total duration of the study. This drug material is an ethanolic extract of *B. monniera* made available by the Central Drug Research Institute, Lucknow. The standardization of this extract was to keep the bacoside content at 55%.

A pre-structured proforma containing details of socio-demographic data, chief complaints, personal history, family history and physical examination was used for documentation. Each subject was evaluated on the Wechsler Memory Scale, revised, Mini-Mental State Examination and for side-effects on Dosage Record Treatment Emergent Symptom Scale (DOTES).^16^ The subtests of Wechsler Memory Scale were used to include general information, orientation, mental control, logical memory, digit forward, digit backward, visual reproduction and paired associated learning, which required an administration period of 30 minutes. A standardized protocol was employed for scoring. The alternate versions of mental control, logical memory, digit forward, digit backward, paired associate learning were used.^17^ Each patient was evaluated on 0, 4, 8, 12 and 16 weeks of the trial. Paired t test was applied to test the significance of observations between the period of visits. The student *t* test was applied to test the significance of observations between the two groups.

## RESULTS

A total of 86 subjects were screened with memory complaints and a tentative diagnosis of AAMI in the outpatient department of Psychiatry, KGMC, Lucknow. Of these, only 40 (37 men, 3 women) subjects fulfilled the inclusion criteria and were randomized to receive either SBME or placebo with 20 subjects in each group. Five subjects (2 in the SBME group and 3 in the placebo group) dropped out and the remaining 35 subjects completed the study. One subject in each group dropped out after the initial visit and 2 subjects in the placebo group and 1 in the SBME group in the 8th week. In the SBME group, the second patient dropped because of maculopapular rashes. No definite reason could be known for other subjects as they did not report back. The sociodemographic and clinical variables are shown in Table 1. The predominant age group was 55–60 years constituting 50% of subjects in the SBME and 65% in the placebo group. There was predominance of men, viz. 95% men in the SBME group and 90% in the placebo group. There was a predominance of subjects educated up to class X–XII, constituting 65% and 55% in the SBME and placebo groups, respectively. Most of the population was urban in both groups, i.e. 90% in the SBME and 95% in the placebo group. All subjects reported an insidious onset of memory complaints. Most subjects in the two groups, i.e. 16 (80%) in the SBME and 17 (85%) in the placebo groups had initially progressive and then stationary course of memory impairment.

**Table 1:** Showing sociodemographic and clinical variables of subjects included in SBME and placebo groups

Variable	SBME		Placebo	
		
	No.	%	No.	%
Age (in years)				
55–60	10	50	13	65
61–65	4	20	5	25
66–70	6	30	2	10
Sex				
Male	10	95	18	90
Female	1	5	2	10
Education				
Below X	0	0	2	10
X–XII	13	65	11	55
Above XII	7	35	7	35
Domicile				
Urban	18	90	19	95
Rural	2	10	1	5
Duration of memory loss (years)				
<2 years	12	60	11	55
>2 years	8	40	9	45

The comparison of Weschler Memory Scale scores of subjects in the SBME and the placebo groups at 0, 4, 8, 12 and 16 weeks is shown in Table 2. The mean±SD on each parameter was compared for drug–drug and 4-weekly time interval observations using the paired *t* test. The baseline variables were found to be comparable in both groups. A significant improvement was observed in the logical memory subtest total score at 4 weeks, and a significant improvement was seen in the mental control, logical memory subtest and paired associate learning score at 8 weeks in both groups. At 12 weeks, besides mental control, logical memory and paired associate learning and total score, the digit forward subtest score also shows a significant improvement with SBME. The placebo group also showed improvement on the mental control, logical memory and paired associate learning score, but this gain in score is highly significant in the SBME group than in the placebo group. Further, there was no memory loss in the total and subtest scores gained between 12 and 16 weeks. The individual subject scores were also evaluated for assessing gain by each subject (Table 3). Table 3 shows the division into two groups depending on achieving improvement of 20% or more. An achievement of >21% improvement is considerable as far as memory parameters are concerned. In the SBME group, 10 subjects (55%) improved by 21% and above and 8 subjects (44.3%) improved up to 20%, whereas in the placebo group none of the subjects showed improvement beyond 20%. This difference in improvement between the two groups was statistically highly significant (more in the SBME group). One subject on placebo developed diarrhoea in the 4th week, the intensity was mild and subsided without medication. Headache was reported by two subjects in the placebo group, which was also mild in intensity and subsided without medication in 5 days. Rashes were reported by one subject in the SBME group in the 8th week. According to the subject, rashes were maculapapular, localized in perioral region and lasted about 15 days. However, at the time of reporting no rashes were seen. No change in clinical and laboratory parameters was observed after the administration of SBME.

**Table 2:** Showing memory subtest scores before (0 week) and after 4, 8, 12 and 16 weeks post drug (values expressed as mean±SD)

Variables	0 Week	4 Week	8 Week	12 Week	16 Week
1. *Mental control*					
SBME	7.4±0.9	7.8±0.7	8.2±0.9	8.6±0.6**	8.6±0.6
Placebo	7.7±0.6	7.7±0.6	8.0±0.4	8.0±0.4	8.0±0.4
*‘t’*		1.94	2.06	3.53	
2. *Logical memory*					
SBME	4.3±1.1	5.9±1.2**	7.4±1.2**	8.7±2.1**	8.8±2.1
Placebo	4.8±1.1	5.5±1.3	6.4±1.8*	6.7±1.6	6.7±1.4
*‘t’*		2.58	2.47	3.48	
3. *Digit forward*					
SBME	6.3±0.5	6.8±0.7	6.6±0.7	7.0±0.7*	7.0±0.4
Placebo	6.8±0.7	6.7±0.7	6.8±0.6	6.8±0.7	6.8±0.6
*‘t’*		1.4	0.55	0.96	
4. *Digit backward*					
SBME	5.5±0.3	5.2±0.4	5.2±0.5	5.2±0.4	5.9±0.8
Placebo	5.7±0.7	5.7±0.8	5.6±0.6	5.7±0.6	5.7±0.6
*‘t’*		0.0	0.35	0.4	
5. *Visual reproduction*					
SBME	11.7±1.6	11.5±1.7	11.6±1.6	11.7±1.7	11.7±1.7
Placebo	12.0±1.9	12.1±1.8	12.0±2.1	12.1±2.2	12.1±2.1
*‘t’*		0.98	0.34	2.48	
6. *Paired associate learn ing*					
SBME	13.9±3.6	14.6±3.3	16.8±2.4**	18.1±2.3**	18.2±2.1
Placebo	12.7±1.4	13.2±1.2	13.7±2.1*	14.5±1.8*	14.5±1.7
*‘t’*		1.26	1.9	2.48	
Total memory score					
SBME	57.4±4.4	60.8±4.7*	66.9±4.7**	70.2±4.8**	70.6±4.0
Placebo	57.5±3.1	59.1±3.4	63.3±4.9*	64.6±4.8*	64.4±4.9
*‘t’*		2.87	10.7	4.98	

* p<0.05

** p<0.01

## DISCUSSION

Central Drug Research Institute, Lucknow has made serious efforts to develop a drug from a traditional herbal plant from Ayurveda, commomly known as Brahmi, which has been claimed to enhance memory. It had already undergone successful evaluation during regulatory and toxicological studies. The present study is a double-blind, placebo-controlled clinical trial designed to evaluate the efficacy of standardized *B. monniera* extract in elderly subjects with AAMI. No effective drug is available in market to treat AAMI. The reason for selecting this group was to study the effects of standardized extract on memory in a mildly deranged clinical situation. In AAMI, the impairment is not very severe and the extract was expected to show improvement in these cases.

Since it was an experimental drug, subjects were registered in the adult outpatient department and a proper follow-up was maintained. Finally, 18 subjects in the SBME and 17 in the placebo group completed the study. The predominant age group in our study was 55–60 years (58.3±5.5 years) constituting 50% in the SBME and 65% (57.7±3.6 years) in the placebo group. This could be because most people in this group are working and their problem of forgetfulness interferes with proper functioning and hence they seek medical advice early. Men outnumber women in both the groups, which could be because predominantly, men are the earning members of the family so they, along with their family, suffer more due to their problem of forgetfulness.

Predominant class of education was X–XII constituting 65% in SBME and 55% in placebo group and majority of the study population was urban which indicates that they are more health conscious, detect their problems early and seek medical help. The analyses of Weschler Memory Scale showed that there are no significant differences in both groups and were comparable at baseline. In personal and current information and orientation test score, there was insignificant change in the two groups at any point of evaluation.

In mental control, there was significant improvement at the end of the 8th week and highly significant at the end of the 12th week which was maintained even after withdrawal of the drug from 13th to 16th week when placebo was given to subjects in the SBME group as well. In the placebo group, significant improvement on mental control occurred only at the end of the 12th week and, even at that stage, there was highly significant difference between the two groups, i.e. higher improvement in the SBME group than in the placebo group. This reflects improvement in the calculating ability of subjects.

In logical memory, there was highly significant improvement in recall of story at the end of the 4th week and onwards in the SBME group, which was maintained even after the withdrawal of drug from 13th to 16th weeks when the placebo was given. In the placebo group, there was significant improvement at the end of the 4th week, which became highly significant at the end of the 8th week and afterwards. Compared with the SBME group, this improvement remained significantly less in the placebo group up to the 12th week.

In the digit forward test, all the subjects enrolled in the study had average baseline values, which ensured that the memory deficit was not due to impairment in attention. There was further improvement in the scores at the end of the 12th week in the SBME group, which was maintained after withdrawal of the drug from 13th to 16th weeks, but the difference was not statistically significant at any point of time of evaluation. It reflects potential for improvement in attention. In digit backward and visual reproduction, the baseline scores were average and there was no significant change at any stage of evaluation in both the groups.

In paired associate learning, the SBME group showed highly significant improvement at the end of 8th week and onwards which was maintained till the end of the 16th week when subjects were given placebo from 13th to 16th weeks. In the placebo group, there was significant improvement at the end of the 8th and 12th week. In contrast, the improvement was highly significant at the end of the 8th and 12th week, showing higher improvement in the SBME group. The between group comparison also showed significant improvement on paired associate learning with SBME than placebo after 8 and 12 weeks of drug administration.

In total score, there was highly significant improvement in both groups from the 4th week onwards and this effect was maintained till the end of 16th week in the SBME group when the drug was withdrawn and the placebo was given to the subjects from 13th to 16th weeks. Difference between the two groups was also statistically significant at the end of the 4th week and became highly significant at the end of 8th week onwards and more so in the SBME group. Table 3 shows that maximum number of subjects, i.e. 10 (55%) in the SBME group, were improved by 21% and above and 8 subjects (44.3%) up to 20% from baseline as compared to the placebo group where all the subjects, i.e. 17, showed improvement only up to 20%. Difference in the improvement between the two groups was statistically significant and SBME was found to be more effective than the placebo.

**Table 3:** Effect of SBME and placebo on AAMI

Percentage improvement (in total Score from 0–12 weeks)	SBME (*n*=18)		Placebo (*n*=17)	
		
	No.	%	No.	%
Up to 20%	8	44.3	17	100
> 21%	10	55.8	0	0

χ^2^ = 13.2 (p<0.01)

Improvement in the placebo group could have been due to learning effects during trial sessions coupled with high expectations from a potential medicine by the subjects. In a similar study carried out at another centre there was highly significant gain in logical memory, digit forward, paired associate learning and total score in the SBME group as compared to the placebo group.^18^

Side-effects were monitored on DOTES. One subject on placebo developed diarrhoea in the 4th week and headache was reported by 2 subjects in the placebo group, which was mild in intensity and subsided quickly. In the SBME group, 1 subject reported rashes in the 8th week. However, at the time of reporting no rashes were seen and had subsided. The subject was taking drug for the past 8 weeks so there could be a possibility of delayed reaction due to the drug and re-challenge was considered, but the subject denied and dropped out of the study. It was difficult to establish the relationship of this side-effect with the drug *per se.* Previous clinical studies have not reported any side-effect in subjects receiving SBME. The safety and tolerability of bacosides A and B has been reported in single and multiple doses in healthy male volunteers.^19^ The detailed pre- and post-drug monitoring of clinical, haematological and biochemical laboratory parameter did not reveal drug-related abnormality.

Thus, it can be concluded that SBME is a relatively safe and effective drug for the treatment of AAMI. However, it needs to be emphasized that small sample included in the study reduced the statistical power and the results therefore are suggestive. Hence, longer duration, large, double-blind studies are needed to confirm these observations.
